# Prognostic Value of *CD1B* in Localised Prostate Cancer

**DOI:** 10.3390/ijerph16234723

**Published:** 2019-11-27

**Authors:** Cheng-Hsueh Lee, Lih-Chyang Chen, Chia-Cheng Yu, Wen-Hsin Lin, Victor C. Lin, Chao-Yuan Huang, Te-Ling Lu, Shu-Pin Huang, Bo-Ying Bao

**Affiliations:** 1 Department of Urology, Kaohsiung Medical University Hospital, Kaohsiung 807, Taiwan; hsueh612@hotmail.com; 2 Graduate Institute of Clinical Medicine, College of Medicine, Kaohsiung Medical University, Kaohsiung 807, Taiwan; 3 Department of Medicine, Mackay Medical College, New Taipei City 252, Taiwan; lihchyang@mmc.edu.tw; 4 Division of Urology, Department of Surgery, Kaohsiung Veterans General Hospital, Kaohsiung 813, Taiwan; ccyu@vghks.gov.tw; 5 Department of Urology, School of Medicine, National Yang-Ming University, Taipei 112, Taiwan; 6 Department of Pharmacy, Tajen University, Pingtung 907, Taiwan; 7 Department of Pharmacy, China Medical University, Taichung 404, Taiwan; wslin@mail.cmu.edu.tw (W.-H.L.); lutl@mail.cmu.edu.tw (T.-L.L.); 8 Department of Urology, E-Da Hospital, Kaohsiung 824, Taiwan; victorlin0098@yahoo.com.tw; 9 School of Medicine for International Students, I-Shou University, Kaohsiung 840, Taiwan; 10 Department of Urology, National Taiwan University Hospital, College of Medicine, National Taiwan University, Taipei 100, Taiwan; cyhuang0909@ntu.edu.tw; 11 Department of Urology, Faculty of Medicine, College of Medicine, Kaohsiung Medical University, Kaohsiung 807, Taiwan; 12 Institute of Biomedical Sciences, National Sun Yat-sen University, Kaohsiung 804, Taiwan; 13 Sex Hormone Research Center, China Medical University Hospital, Taichung 404, Taiwan; 14 Department of Nursing, Asia University, Taichung 413, Taiwan

**Keywords:** CD antigen, prostate cancer, biomarker, prognosis, *CD1B*

## Abstract

Cluster of differentiation (CD) antigens are cell surface markers used to differentiate haematopoietic cell types. These antigens are present in various malignancies and are reportedly linked to patient prognosis; however, they have not been implemented as prostate cancer progression markers. Here, we aimed to assess the impact of genetic variation in haematopoietic cell CD markers on clinical outcomes in patients with prostate cancer. An association study of 458 patients with prostate cancer was conducted to identify single-nucleotide polymorphisms in 11 candidate CD marker genes associated with biochemical recurrence (BCR) after radical prostatectomy. Identified predictors were further evaluated in an additional cohort of 185 patients. Joint population analyses showed that *CD1B* rs3181082 is associated with BCR (adjusted hazard ratio 1.42, 95% confidence interval 1.09–1.85, *p* = 0.010). In addition, rs3181082 overlapped with predicted transcriptional regulatory elements and affected *CD1B* expression. Furthermore, low *CD1B* expression correlated with poorer BCR-free survival. Our results indicated that *CD1B* rs3181082 confers prostate cancer progression and may help improve clinical prognostic stratification.

## 1. Introduction

Prostate cancer is one of the most prevalent cancers in men worldwide. Although prostate cancer incidence and mortality are lower in most Asian countries as compared to those in other regions, they are rapidly increasing with the implementation of modern screening methods and the Westernisation of diet and lifestyle [1]. At present, prostate cancer diagnosis relies mainly on prostate-specific antigen (PSA) detection, digital rectal examination, and magnetic resonance imaging. Unfortunately, these conventional measures are suboptimal, especially for the diagnosis of intermediate-risk prostate cancer, resulting in unnecessary biopsies and overtreatment for men with low-risk prostate cancer [2]. There are now more than 100 prostate cancer susceptibility loci that have been identified by genome-wide association studies and have been thought to explain 30% of the disease heritability [3]. A recently developed diagnostic tool that combines plasma protein biomarkers, single nucleotide polymorphisms (SNPs), and clinical variables, showed a better performance [area under the curve (AUC) = 0.74] to detect high-risk prostate cancer compared with PSA alone (AUC = 0.56), and avoid 44% of benign biopsies [4]. Therefore, the integration of germline biomarkers with clinical predictors might help distinguish between patients at increased risk for lethal prostate cancer and those with indolent tumours.

Proteins located on the cell membrane are of particular interest as therapeutic targets. Membrane proteins direct several pivotal biological processes, including cell–cell communication, cell proliferation, cell motility and adhesion, and responses to external stimuli. These membrane proteins, also referred to as surface markers, are commonly detected through a cluster of differentiation (CD) designation. CD markers are widely used in lineage studies of haematopoietic cell types. Recent findings have shown that leukaemia and several solid tumours contain a rare subpopulation of cells with specific surface CD markers that has the potential to self-renew, sustain cancer growth, and predict prognosis [5,6]. However, it remains unclear whether genetic variants and expression patterns of CD markers could be used to identify clinically distinct cancer cell types and predict patient outcomes. In the present study, we investigated the associations of genetic variants in 11 key haematopoietic cell CD marker genes with biochemical recurrence (BCR) in patients with localised prostate cancer after radical prostatectomy (RP). Further functional analyses supported the involvement of *CD1B* in prostate cancer progression.

## 2. Materials and Methods

### 2.1. Patient Recruitment and Data Collection

A total of 643 patients with histopathologically confirmed prostate cancer who underwent RP were recruited from three Taiwan medical centres, Kaohsiung Medical University Hospital, Kaohsiung Veterans General Hospital, and National Taiwan University Hospital, as described previously [7]. Patients were randomly divided into discovery and replication sets at a 7:3 ratio. The discovery set, consisting of 458 patients, was used to assess the association between haematopoietic cell lineage marker gene variants and BCR, and the replication set, consisting of 185 patients, was used to validate the prognostic value of positive SNPs. This study received approval from the institutional review board of Kaohsiung Medical University Hospital (IRB no: KMUHIRB-2013132), and all participants provided written informed consent according to institutional guidelines. The clinicopathologic information was obtained through a medical chart review. BCR was defined as two consecutive PSA measurements of 0.2 ng/mL or greater after RP [8,9,10,11]. BCR-free survival was calculated from the date of RP to the date of BCR.

### 2.2. Single Nucleotide Polymorphism (SNP) Selection and Genotyping

According to the literature review, a total of 11 cancer–related genes in the Kyoto Encyclopedia of Genes and Genomes (KEGG) haematopoietic cell lineage pathway, CD19 molecule (*CD19*) [12], CD1b molecule (*CD1B*) [13], CD1c molecule (*CD1C*) [14], CD3d molecule (*CD3D*) [15], CD3e molecule (*CD3E*) [15], CD3g molecule (*CD3G*) [15], CD5 molecule (*CD5*) [16], CD8a molecule (*CD8A*) [17], fms-related tyrosine kinase 3 (*FLT3*, also known as *CD135*) [18], integrin subunit alpha 5 (*ITGA5*, also known as *CD49E*) [19], and membrane spanning 4-domains A1 (*MS4A1*, also known as *CD20*) [20], were included in this study. Initially, 19 haplotype-tagging SNPs (htSNPs) in these genes were selected using SNPinfo [21] based on the following criteria: a minor allele frequency of >0.05 in the HapMap CHB (Han Chinese in Beijing) population, a pairwise linkage disequilibrium (*r*^2^) of >0.8, potentially functional, and a maximum of five htSNPs per gene. Genomic DNA was isolated from peripheral blood using the QIAamp DNA Blood Mini Kit (Qiagen, Valencia, CA, USA) according to the manufacturer’s instructions and genotyped using Agena Bioscience iPLEX matrix-assisted laser desorption/ionisation time-of-flight mass-spectrometry technology at the National Centre for Genome Medicine, Taiwan, as described previously [22]. The average genotype call rate for these SNPs was 97.7%, and the average concordance rate was 99.9% among 35 blind duplicated quality control samples. One htSNP in *MS4A1* that did not conform to the Hardy–Weinberg equilibrium (*p* < 0.05) was excluded, leaving a total of 18 htSNPs for further analyses.

### 2.3. Bioinformatics Analysis

The regulatory annotation of *CD1B* rs3181082 and correlated variants (*r*^2^ ≥ 0.8 in East Asians from the 1000 Genomes Project) was conducted using HaploReg v4.1 [23]. An expression quantitative trait loci (eQTL) analysis was performed using the Genotype-Tissue Expression (GTEx) portal [24]. The prognostic significance of *CD1B* in prostate cancer was analysed using the publicly available GSE40272 [25] and GSE16560 [26] microarray datasets.

### 2.4. Statistical Analysis

Associations for different genotypes or gene expression groups were evaluated using Kaplan–Meier analyses with log-rank tests. Univariate and multivariate Cox regression analyses were used to calculate the crude and adjusted hazard ratios (HRs) and their 95% confidence intervals (CIs) without or with adjustment for age, PSA at diagnosis, pathologic Gleason score, stage, and lymph node metastasis. A meta-analysis was performed to combine the normalised effect size of the eQTLs among 10 tissues with >200 samples in the GTEx database. All statistical analyses were performed using Statistical Package for the Social Sciences software version 19.0.0 (IBM, Armonk, NY, USA). A two-sided *p*-value of <0.05 was considered statistically significant.

## 3. Results

Detailed clinicopathologic characteristics of participants in the discovery and replication sets are presented in Table S1. No significant differences in clinicopathologic features were observed between the discovery and replication sets. PSA at diagnosis, pathologic Gleason score, stage, and lymph node metastasis were significantly associated with BCR after the median follow-up times of 54 and 74 months in the discovery and replication sets, respectively.

**Figure 1:**
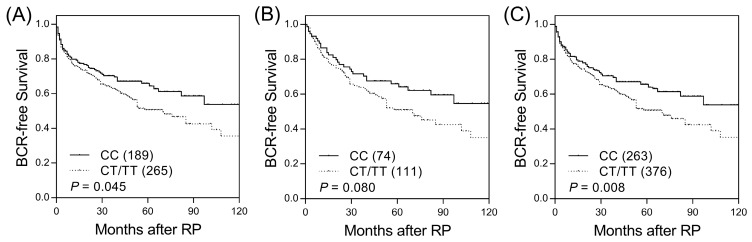
Kaplan–Meier survival curves for biochemical recurrence-free survival according to *CD1B* rs3181082 genotypes in the (**A**) discovery set, (**B**) replication set, and (**C**) combined analysis. Numbers in parentheses indicate the number of patients.

Among the 18 SNPs in candidate haematopoietic cell lineage marker genes, two SNPs (*CD1B* rs3181082 and *CD1C* rs76926515) were associated with BCR-free survival (*p* ≤ 0.045, Table S2 and Figure 1A) and were further evaluated in an independent replication set. *CD1B* rs3181082 showed a marginal association with BCR (*p* = 0.080, Table 1 and Figure 1B) and consistent trends in the discovery and replication sets. In the combined sample set, the risk of BCR was higher for patients with the *CD1B* rs3181082 T allele than for patients with the CC genotype (HR 1.40, 95% CI 1.09–1.80, *p* = 0.009, Table 1 and Figure 1C). This association remained significant (*p* = 0.010) in multivariate analysis after adjusting for other risk covariates, including age, PSA at diagnosis, pathologic Gleason score, stage, and lymph node metastasis. However, *CD1C* rs76926515 did not improve prediction beyond the clinical features to influence BCR (*p* = 0.090).

**Figure 2:**
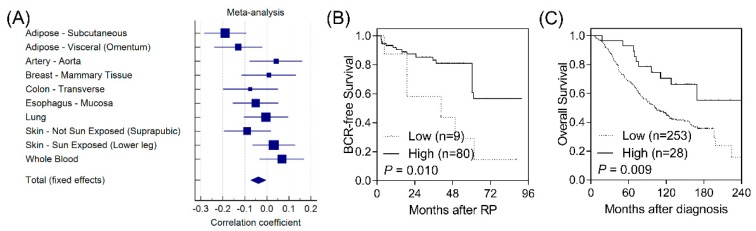
Functional analyses of *CD1B* rs3181082. (**A**) Forest plot represents the correlation between rs3181082 and *CD1B* expression in tissues with >200 samples and genotype data from the Genotype-Tissue Expression (GTEx) dataset. Decreased expression of *CD1B* is associated with poor biochemical recurrence-free survival (**B**) and overall survival (**C**) in patients with prostate cancer. Patients were classified into low- and high-risk groups by an optimisation algorithm for the minimum *p*-value. Numbers in parentheses indicate the number of patients.

According to functional analyses using HaploReg, *CD1B* rs3181082 overlapped with promoter and enhancer histone marks and DNase hypersensitivity sites and was predicted to alter the NF-κB (nuclear factor kappa B) motif (Table S3). These results suggested that rs3181082 influences *CD1B* gene expression. We performed an eQTL analysis to evaluate the effect of rs3181082 on *CD1B* using the GTEx dataset. As shown in Figure 2A, there was a trend toward decreased *CD1B* expression in rs3181082 T allele carriers (*p* = 0.024) in the meta-analysis of 10 tissues with >200 samples in GTEx.

To further confirm the biological functions of *CD1B* during prostate cancer progression, we investigated the correlation of *CD1B* expression with disease outcomes in publicly available microarray datasets. Low *CD1B* expression was consistently associated with a poorer BCR in GSE40272 (*p* = 0.010, Figure 2B) and with overall survival in GSE16560 (*p* = 0.009, Figure 2C).

## 4. Discussion

Despite recent advancements in multimodal therapies for prostate cancer, the identification of patient subsets that will benefit from such treatments remains a challenge. In this study, using two-stage genetic association studies, we identified *CD1B* rs3181082 as an independent prognostic factor related to disease recurrence in patients with prostate cancer. Further functional analyses showed that rs3181082 influences *CD1B* expression, which in turn correlated with patient prognosis.

rs3181082 is located 1.2 kb upstream of the *CD1B* promoter region and is predicted to overlap with an NF-κB binding site. In our functional analyses, the rs3181082 T allele was associated with the down-regulation of *CD1B* in various tissues. CD1B is a member of the Group 1 CD1 family of transmembrane glycoproteins, which are related to major histocompatibility complex class I-like molecules. CD1 molecules mediate the presentation of a wide array of self- and foreign-lipid antigens to T-cell receptors on T cells [27]. CD1B exhibits the largest antigen binding groove among CD1 molecules, allowing it to present a wide array of lipid antigens [28]. Recent studies have indicated that normal cells have distinct expression patterns of CD1 molecules compared to tumour cells, including prostate cancer [29,30]. More importantly, the upregulation of lipid species with modified structures during oncogenesis generates self-lipid antigens that are presented by CD1 molecules and can be visible to the immune system by activating the cognate T cells [31]. Indeed, it has been shown that CD1B-restricted self-lipid reactive T cells respond more potently to tumour-derived phospholipids than lipids extracted from normal cells, and the adoptive transfer of these T cells into mice harbouring CD1B-expressing lymphoma results in tumour control [13]. These data demonstrated the anti-tumour potential of CD1B-autoreactive T cells and their potential application in adoptive immunotherapy. In addition, increasing evidence has shown that tumour cells frequently recruit myeloid cells to the tumour microenvironment [32]. Myeloid cells can exert antitumour functions by eradication of the malignant cells in a context-dependent manner, but they can also be moulded by tumour cells into immunosuppressive tumour-infiltrating myeloid cells that support cancer progression [33]. Since *CD1B* is abundantly expressed on the myeloid cells, it is possible that rs3181082 or other untyped functional SNPs in linkage disequilibrium have a modest effect on the function of *CD1B*, which in turn has an impact on tumour immunity and clinical outcomes in patients with prostate cancer. Besides prostate cancer, CD1B is also expressed in thyroid, liver, colorectal, breast, urothelial, and stomach cancers according to the Human Protein Atlas database. However, the prognostic value of *CD1B* rs3181082 in those malignancies is still unclear and needs to be further investigated.

The sample sizes of the discovery (n = 458) and replication (n = 185) sets might not have sufficient statistical power to detect SNPs that confer moderate changes in risk. There was only a marginal association between *CD1B* rs3181082 and BCR in the replication cohort (*p* = 0.080). Furthermore, the retrospective nature of this study allowed us to include only cases from the Taiwanese population; additional studies using a prospective design, larger sample size, and multi-ethnic cohorts might help validate our findings.

## 5. Conclusions

Our results indicate that *CD1B* rs3181082 correlates with prostate cancer outcomes and may provide information for the improved prediction of prostate cancer progression and guide disease monitoring. Further studies of *CD1B* rs3181082 may identify new biological pathways affecting postoperative progression in prostate cancer.

## Figures and Tables

**Table 1:** SNPs associated with BCR in patients with prostate cancer receiving RP.

Gene SNP	Discovery			Replication			Combined				
Genotype	*N*	BCR	*P*	*N*	BCR	*P*	MST, Months	HR (95% CI)	*P*	HR (95% CI) ^a^	*P* ^a^
*CD1B* rs3181082											
CC	189	63	0.053	74	30	0.150	127	1.00		1.00	
CT	207	88		85	46		70	**1.39 (1.07–1.81)**	**0.015**	**1.41 (1.06–1.86)**	**0.018**
TT	58	29		26	14		58	1.42 (0.99–2.04)	0.058	1.46 (1.00–2.12)	0.050
CT/TT vs. CC			**0.045**			0.080		**1.40 (1.09–1.80)**	**0.009**	**1.42 (1.09–1.85)**	**0.010**
TT vs. CC/CT			0.314			0.692		1.19 (0.86–1.64)	0.308	1.21 (0.86–1.69)	0.270
*CD1C* rs76926515											
AA	359	135	0.155	151	69	0.421	102	1.00		1.00	
AG	37	20		31	21		45	**1.74 (1.25–2.44)**	**0.001**	**1.47 (1.03–2.11)**	**0.036**
GG	3	0		3	0		–	–		–	
AG/GG vs. AA			**0.030**			0.136		**1.52 (1.09–2.13)**	**0.014**	1.37 (0.95–1.96)	0.090

Abbreviations: SNP, single nucleotide polymorphism; BCR, biochemical recurrence; MST, median BCR-free survival time; RP, radical prostatectomy; HR, hazard ratio; CI, confidence interval. ^a^ Adjustment for age, PSA at diagnosis, pathologic Gleason score, stage, and lymph node metastasis. *p* < 0.05 are in boldface.

## Supplementary Materials

The following are available online at https://www.mdpi.com/1660-4601/16/23/4723/s1: Table S1: Clinicopathologic characteristics of the study populations, Table S2: Genotyped SNPs and the *p* values of their association with BCR after RP, Table S3: Regulatory annotation of variants linked with *CD1B* rs3181082.
