# Supplementary material for: Prognostic Value of CD1B in Localised Prostate Cancer
Source: Int J Environ Res Public Health. 2019 Nov 27;16(23):4723. doi: 10.3390/ijerph16234723 (PMC6926967; doi:10.3390/ijerph16234723)
Supplement: Supplementary file 1 [file ijerph-16-04723-s001.pdf]

Table S1. Clinicopathologic characteristics of the study populations

Characteristics	Discovery	Replication
N of patients	458	185
Age at diagnosis		
Median, years (IQR)	66 (61-70)	66 (61-70)
PSA at diagnosis, *N* (%)		
Median, ng/mL (IQR)	11.1 (7.1-17.5)	11.0 (6.9-18.7)
≤ 20	347 (79.0)	131 (76.2)
>20	92 (21.0)	41 (23.8)
Pathologic Gleason score, *N* (%)		
2-6	160 (35.3)	72 (39.8)
7-10	293 (64.7)	109 (60.2)
Pathologic stage, *N* (%)		
1	59 (13.1)	25 (13.9)
2	247 (54.9)	102 (56.7)
3	134 (29.8)	47 (26.1)
4	10 (2.2)	6 (3.3)
Lymph node metastasis, *N* (%)		
Negative	433 (95.6)	178 (97.8)
Positive	20 (4.4)	4 (2.2)
Biochemical recurrence, *N* (%)	184 (40.2)	90 (48.6)
Median follow-up, months	54	74

Abbreviations: IQR, interquartile range; PSA, prostate-specific antigen.

Table S2. Genotyped SNPs and the *P* values of their association with BCR after RP

Gene	SNP ID	Chromosome	Position	MAF	HWE	BCR		
						Additive	Dominant	Recessive
CD19	rs2070961	16	28938152	0.211	0.751	0.878	0.231	–
CD1B	rs3181082	1	158332703	0.353	1.000	0.053	**0.045**	0.314
CD1C	rs76926515	1	158273263	0.054	0.204	0.155	**0.030**	–
CD3D	rs3212264	11	118345519	0.445	0.810	0.171	0.385	0.164
CD3E	rs7928058	11	118300524	0.270	0.301	0.201	0.185	0.581
CD3E	rs7480736	11	118301649	0.470	0.896	0.114	0.136	0.275
CD3E	rs2231440	11	118304724	0.198	0.676	0.569	0.487	–
CD3G	rs1561966	11	118350759	0.436	0.962	0.429	0.883	0.206
CD5	rs616340	11	61108829	0.081	0.354	0.546	0.470	–
CD8A	rs1051386	2	86784950	0.182	0.382	0.626	0.438	–
CD8A	rs13023213	2	86794820	0.074	0.523	0.336	0.367	–
FLT3	rs2491218	13	28029904	0.259	0.381	0.202	0.243	0.428
FLT3	rs2491239	13	28039007	0.139	0.762	0.101	0.146	–
FLT3	rs1933437	13	28050157	0.240	0.819	0.276	0.287	0.577
FLT3	rs9554229	13	28065112	0.164	0.222	0.840	0.920	–
FLT3	rs1320534	13	28067397	0.186	0.112	0.416	0.695	–
ITGA5	rs1270919	12	54410188	0.105	0.830	0.829	0.781	–
MS4A1	rs4939362	11	60455545	0.353	0.770	0.882	0.658	0.713

Abbreviations: SNP, single nucleotide polymorphism; BCR, biochemical recurrence; RP, radical prostatectomy; MAF, minor alleles frequency; HWE, Hardy-Weinberg equilibrium.

P values for log-rank test.

$P < 0.05$ is in boldface.

Table S3. Regulatory annotation of variants linked with *CD1B* rs3181082

Chromosome	Position	LD (r^2)	SNP ID	Reference allele	Alternate allele	ASN frequency	Promoter histone marks	Enhancer histone marks	DNAse	Proteins bound	Motifs changed	GRASP QTL hits	Selected eQTL hits	dbSNP function annotation
1	158264494	0.81	rs80312173	T	C	0.35					Bcl6b,HDAC2,Ik-2,Mef2			6.2kb 3' of CD1A
1	158268712	0.81	rs201631500	CGGGGGAGGGGG	C	0.38					AP-1,CAC-binding-protein,CCNT2,CHD2,E2F,EWSR1-FLI1,Egr-1,Ets,MAZ,MZF1::1-4,Myc,NRSF,PU.1,Pax-4,Pou2f2,RREB-1,SP1,STAT,Sp4,TATA,TFII-I,UF1H3BETA,VDR,WT1,YY1,ZNF219,ZNF263,Zfp281,Zfp740			10kb 3' of CD1A
1	158275041	0.87	rs61818716	C	G	0.36		BLD, THYM	ESDR,IPSC,BLD,BLD,BLD		Mxi1,RFX5			15kb 5' of CD1C
1	158277108	0.93	rs41516545	T	C	0.37								13kb 5' of CD1C
1	158280010	0.93	rs12565919	T	A	0.36		BLD			CEBPB,CEBPD			9.8kb 5' of CD1C
1	158293976	0.95	rs3138107	A	T	0.37		BLD, THYM			HDAC2,Pou1f1,Pou2f2,Pou5f1			CD1C 3'-UTR
1	158294366	0.95	rs77773003	G	A	0.37		BLD			NF-E2			CD1C 3'-UTR
1	158313118	0.95	rs77702869	TC	T	0.37		BLD			Foxa,GATA,HDAC2,Hlx1,Ik-2,Nanog,Nkx6-1,Pou2f2,Sox,XBP-1			15kb 3' of CD1B
		0.94	rs150645844	A	G	0.36					CDP,Hbp1,Sox,TCF4			12kb 3' of CD1B
1	158318802	0.82	rs146320976	C	T	0.34					GR			9.1kb 3' of CD1B
1	158319920	0.93	rs61818723	C	T	0.37					Nanog			8kb 3' of CD1B
1	158324728	1	rs16840093	A	C	0.37	BLD	BLD			CIZ,PPAR		1 hit	3.2kb 3' of CD1B
1	158325219	1	rs1321646	C	T	0.37		BLD				1 hit	1 hit	2.7kb 3' of CD1B
1	158328170	1	rs16840096	C	T	0.37	THYM, BLD	BLD			AIRE,Evi-		1 hit	CD1B 3'-UTR

										1,Foxm1,HNF6,Pbx-1,Pbx3			
1	158330516	1	rs962879	G	C	0.37	THYM, BLD	BLD	THYM	SZF1-1	1 hit	1 hit	CD1B intronic
1	158332703	1	**rs3181082**	C	T	0.37	THYM, BLD	BLD		NF-kappaB		1 hit	1.2kb 5' of CD1B
1	158336540	0.97	rs61818724	C	G	0.38		BLD	ESC,IPSC	E2A,NF-Y			5kb 5' of CD1B
1	158336678	0.99	rs61818725	T	C	0.37		BLD		Hsf			5.1kb 5' of CD1B
1	158338893	0.99	rs12566915	C	G	0.37				Pbx3,Zec			7.4kb 5' of CD1B
1	158339625	0.99	rs114638986	G	A	0.37			IPSC				8.1kb 5' of CD1B
1	158340837	0.95	rs145921349	A	G	0.37				CDP,DMRT5,DMRT7			9.3kb 5' of CD1B
1	158341489	0.97	rs143007912	C	T	0.37				E2F,Irf,Pbx3,SP1			10kb 5' of CD1B
1	158341998	0.98	rs76638901	T	C	0.37				Brachyury,GR,Pax-4,Pax-8,RBP-Jkappa			10kb 5' of CD1B
1	158346134	0.91	rs2317955	G	T	0.37				BRCA1,Fox,Foxa,Foxd3,Foxi1,Foxj1,Foxj2,Foxl1,Foxp1,GR,Pou5f1,RREB-1		1 hit	7.3kb 5' of CD1E
1	158346846	0.91	rs61818727	A	T	0.37							6.6kb 5' of CD1E
1	158357108	0.87	rs61818738	T	C	0.37	THYM		THYM	Foxp3,Hand1,Smad3,Smad			CD1E 3'-UTR
